# Review of the Reporting of Survival Analyses within Randomised Controlled Trials and the Implications for Meta-Analysis

**DOI:** 10.1371/journal.pone.0154870

**Published:** 2016-05-05

**Authors:** Sarah Batson, Gemma Greenall, Pollyanna Hudson

**Affiliations:** DRG Abacus, Bicester, United Kingdom; University of Florence, ITALY

## Abstract

**Background:**

Meta-analysis is a growing approach to evidence synthesis and network meta-analysis in particular represents an important and developing method within Health Technology Assessment (HTA). Meta-analysis of survival data is usually performed using the individual summary statistic—the hazard ratio (HR) from each randomised controlled trial (RCT).

**Objectives:**

The objectives of this study are to: (i) review the methods and reporting of survival analyses in oncology RCTs; and (ii) assess the suitability and relevance of survival data reported in RCTs for inclusion into meta-analysis.

**Methods:**

Five oncology journals were searched to identify Phase III RCTs published between April and July 2015. Eligible studies included those that analysed a survival outcome.

**Results:**

Thirty-two RCTs reporting survival outcomes in cancer populations were identified. None of the publications reported details relating to a strategy for statistical model building, the goodness of fit of the final model, or final model validation for the analysis of survival outcomes. The majority of studies (88%) reported the use of Cox proportional hazards (PH) regression to analyse survival endpoints. However, most publications failed to report the validation of the statistical models in terms of the PH assumption.

**Conclusions:**

This review highlights deficiencies in terms of reporting the methods and validity of survival analyses within oncology RCTs. We support previous recommendations to encourage authors to improve the reporting of survival analyses in journal publications. We also recommend that the final choice of a statistical model for survival should be informed by goodness of model fit to a given dataset, and that model assumptions are validated. The failure of trial investigators and statisticians to investigate the PH for RCT survival data is likely to result in clinical decisions based on inappropriate methods. The development of alternative approaches for the meta-analysis of survival outcomes when the PH assumption is implausible is required if valid clinical decisions are to be made.

## Introduction

Survival analysis is important in the assessment of the efficacy of interventions. Oncology represents a major disease area where survival analysis is a fundamental aspect of clinical management and drives decision-making around treatment options. Time to event data are captured when the time elapsing before a particular event is of interest. Such data are generically described as survival data (the time survived until an event). As time to event data are rarely normally distributed, their use with conventional statistical methods is inappropriate. For example, most patients in a given population might experience an event early on, but some will not experience the event for a longer period of time over the course of a trial and beyond. Survival data derived from clinical trials are usually presented in plot form using Kaplan Meier (KM) estimates of survival. Kaplan Meier plots can be used to approximate measures such as median survival times [1]. The hazard of an event based on the survival data is usually estimated using statistical modelling. The Cox proportional hazards (PH) model represents by far the most common model applied to the analysis of time to event outcomes in randomised controlled trials (RCTs). The Cox PH model is a semi-parametric model that does not make any assumptions about the shape of the underlying hazard function but does assume that the hazard rates for treatment groups are proportional over time. Additional methods of survival analysis include the non-parametric log-rank test to compare treatment groups, and parametric PH models for which the hazard is assumed to follow a specific statistical distribution, such as the Weibull, exponential, and Gompertz distributions. Survival analysis is of particular importance in clinical oncology as the majority of cancer studies investigate time to event endpoints—commonly overall survival (OS) and progression-free survival (PFS).

Network meta-analysis (NMA) is a growing approach to evidence synthesis that allows the synthesis of all available evidence from an extensive evidence network and, in a single analysis, the estimation of the efficacy of each treatment compared with all comparators. Network meta-analysis represents an important and developing method within Health Technology Assessment (HTA). Network meta-analyses and indirect comparisons are acknowledged methodologies by HTA agencies worldwide including the National Institute for Health and Care Excellence (NICE), the Canadian Agency for Drugs and Technologies in Health (CADTH), the French Haute Autorité de la Santé (HAS), and the Pharmaceutical Benefits Advisory Committee (PBAC) in Australia, as well as emerging national agencies in Austria, Brazil, Colombia, Cuba, and Ireland [2]. Meta-analysis of survival data is usually performed using the individual summary statistic—the hazard ratio (HR) from each study as a measure of relative treatment effect. This approach assumes that the PH assumption holds—that is, the relative hazards of the event are constant over time. Study level data, obtained from trial publications for use in meta-analysis are taken at face value as access to the individual participant data (IPD) is rarely available. Where estimates of relative treatment effect are based on statistical models of survival data that do not account for violations of the PH assumption, both the study results and subsequent meta-analyses will be biased. When the PH assumption is imposed on multiple studies in an NMA, this can lead to substantial bias to the point where interpretation of the results requires extreme caution. The primary outcome in cost-effectiveness analyses for drug reimbursement is often the differences in the survival between interventions and therefore the implausibility of the PH assumption can impact decisions based upon cost-effectiveness analyses.

The current review was performed to assess the application, reporting, and adequacy of survival analyses in oncology Phase III RCTs in order to potentially inform future meta-analyses and improve the quality of decision-making.

## Methods

The review was restricted to studies in the oncology setting because it represents a major disease area where survival analysis typically drives decisions around treatment options.

### Search strategy

Electronic databases were searched on 28 August 2015 (Embase; Ovid MEDLINE^®^ In-Process & Other Non-Indexed Citations; Ovid MEDLINE^®^)[S1 Table, supporting information]. Two reviewers independently screened the titles and abstracts of identified citations using pre-specified eligibility criteria. Potentially relevant citations were then screened based on the full publication to identify definite studies for inclusion. Disagreements were resolved through discussion until a consensus was reached, or via the involvement of a third reviewer when necessary.

The inclusion and exclusion criteria are summarised in Table 1.

**Table 1:** Inclusion and exclusion criteria.

	Include	Exclude
**Study design**	Phase III RCTs	Phase I/II RCTs, observational studies, and reviews/editorials.
**Disease/population**	No restriction	No restriction
**Intervention**	No restriction	No restriction
**Outcomes**	Kaplan-Meier or actuarial survival curves, log-rank or similar tests, and parametric or semi-parametric survival analyses.	Publications not reporting a survival analysis
**Journal**	CA: A Cancer Journal for Clinicians, the Lancet Oncology, Journal of Clinical Oncology, Cancer Discovery, and the Journal of the National Institute of Cancer.	All other journals
**Publication type**	Full publications	Abstracts
**Country**	No restriction	No restriction
**Language**	English publications	Non-English publications (including those with an English abstract)
**Year of publication**	April to July 2015	Pre-April 2015

Abbreviations: RCT, randomised controlled trial.

### Data extraction

Data extraction was performed into an extraction template and verified by a second extractor. Disagreements were discussed with a third party. Information extracted from included studies included: sample size, follow up time, study end points, explanatory variables included in the model, details of graphical presentation of survival analyses, details of univariate or multivariate analysis methodology and results presentation, details of subset analyses, and the use of statistical software.

## Results

### Overview and sample size

**Fig 1:**
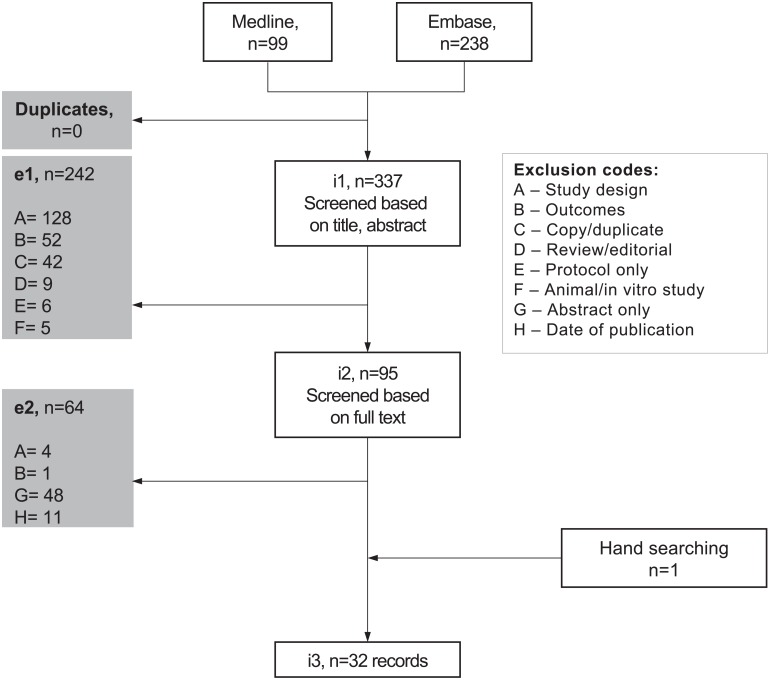
Study flow diagram.

In total, 32 publications of Phase III RCTs from the Journal of Clinical Oncology, the Journal of the National institute of Cancer and Lancet Oncology were included in the review [3–34]. The study flow diagram is shown in Fig 1. A completed PRISMA checklist can be found in S2 Table, supporting information. The publications detailed RCTs in a range of types of cancer populations, which included but were not limited to those with Hodgkin lymphoma, breast, cervical, gastric, head and neck, prostate and lung cancers.

Total intention-to-treat (ITT) sample sizes ranged from 107 [13] to 2,716 patients [8]. All publications analysed the ITT populations except one publication which used the ‘analyzable population’, which excluded a single patient who withdrew and 48 additional patients who were determined ineligible for study inclusion at baseline [8]. The number of events was reported for at least one survival endpoint in 23 of the publications [3–6, 8–12, 14–16, 20–25, 27–29, 31, 33] and was reported for all survival endpoints analysed in half of the publications [3–6, 8, 9, 11, 14–16, 20, 21, 24, 25, 28, 29, 33]. Eighteen publications reported subgroup analyses [3–7, 9, 10, 12, 14, 16, 17, 20, 29–34], many of which reported that the sub-populations analysed were based on pre-specified stratification factors. The use of statistical computing software was reported in 17 publications [3, 6, 7, 9, 10, 13, 14, 17, 18, 20, 22–24, 26, 27, 29, 31], and included SPSS (n = 14), SAS (n = 3), R (n = 1), and other packages (n = 3). Three publications reported the use of more than one software package [14, 22, 26].

### Endpoints

**Fig 2:**
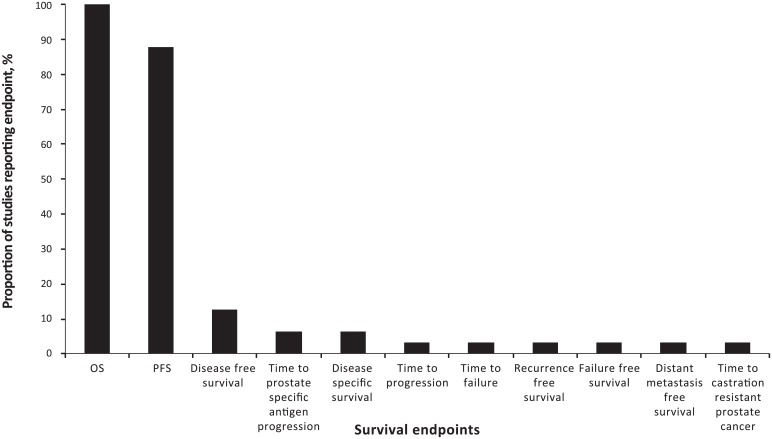
Bar graph summarising the survival endpoints reported across the studies.

The number of survival endpoints analysed in each publication ranged from 1–6 (median of 2). All publications reported the analysis of OS, of which twenty-two publications also reported PFS [3, 6, 7, 10–16, 18, 20, 22, 23, 25–27, 29, 31–34]. Other survival outcomes analysed in the identified studies included failure-free survival, time to progression, disease-free survival, time to prostate-specific antigen progression, recurrence-free survival, distant metastasis-free survival, time-to-castration-resistant prostate cancer, time to failure and disease-specific survival (Fig 2). Endpoints were not defined consistently for all outcomes in each publication although most studies (n = 27) defined at least one survival outcome. In 21 publications, all analysed survival endpoints were defined [3, 4, 7, 8, 10, 12, 14–22, 25, 27, 29, 31, 32, 34]. Five publications did not define any survival endpoint [5, 11, 24, 26, 30], but in three of these publications survival endpoints were described as secondary outcomes [24, 26, 30].

Overall survival was consistently defined as time-to-death from any cause, and PFS was consistently defined as time to progression or death from any cause. Four of 22 publications reporting PFS stated that progression was determined by Response Evaluation Criteria in Solid Tumours (RECIST) [12, 13, 16, 25], and an additional two publications detailed progression as radiographic [25, 34].

### Follow-up

Twenty-eight publications reported the start and end of accrual dates [3–10, 12–16, 18–20, 22–24, 26–34] and sixteen of these also reported the date for the end of follow-up (cut-off point for the analyses) [3, 5, 6, 8, 10, 12, 14, 16, 19, 20, 23, 24, 27, 29, 32, 34]. Twenty-nine publications reported a measure of follow-up time [3, 4, 6–14, 16–29, 31–34], the majority of which were in the form of median follow-up (n = 25). Three publications failed to report a measure of follow-up and, in each case, the events of interest for each outcome were not reported for all patients [15, 17, 25]. The method of calculating a follow-up measure was rarely reported or was inconsistent; examples included calculation of follow-up for ‘patients with an event’ and for ‘patients still alive’.

### Statistical methods

**Fig 3:**
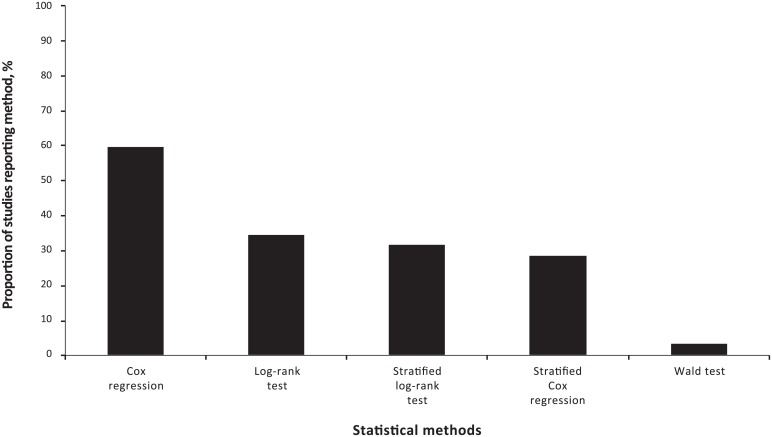
Bar graph summarising the statistical methods used across the studies.

Nineteen publications reported univariate analyses [3–6, 8–10, 13, 14, 17, 19, 21, 22, 24, 26–28, 30, 34] and nineteen publications reported multivariate analyses [3, 4, 6, 7, 9, 11–13, 15–17, 19, 20, 22, 23, 29, 31, 32, 33]. Eight publications reported both univariate and multivariate analyses for the same endpoints [3, 4, 6, 9, 13, 17, 19, 22]. The reported statistical methods for comparing treatment groups included the log-rank test (n = 11) [3–5, 8, 13, 14, 19, 22, 24, 26, 27], stratified log-rank test (n = 10) [7, 9, 11, 12, 15, 20, 23, 29, 31, 32], the Wald test (n = 1) [30], Cox regression analyses (n = 19) [3, 4, 6, 8–10, 13, 14, 17, 19, 22–24, 26–30, 34], and stratified Cox regression analyses (n = 9) [7, 11, 16, 18, 20, 25, 31–33] (Fig 3).

Across publications reporting only univariate analyses [5, 8, 10, 14, 21, 24, 26–28, 30, 34], there were generally two statistical methods reported: the log-rank test to compare treatment groups and generate p-values, and Cox regression to estimate hazard ratios (HRs) and their associated confidence intervals (CIs). Across the multivariate analyses the number of additional variables in the Cox regression models or stratification factors ranged from one to ten. The rationale for the choice of variables in the multivariate models or stratification factors was rarely reported, but three publications did report the use of predefined stratification factors [11, 20, 23]. Continuous outcomes were categorised for use as dichotomous variables in multivariate models or subgroup analyses across 18 of the identified publications [3, 4, 7, 14, 18, 20–28, 30, 31, 33, 34], but the rationale for the choice of cut-off for dichotomisations was not provided in any of the publications.

### Cox proportional hazards model

The Cox PH model was reported in 28 publications as either a univariate or multivariate analysis [3, 4, 6–11, 13, 14, 16–20, 22–34]. Notably, no other statistical models were reported for the analysis of survival outcomes. In terms of Cox regression analyses, none of the studies reported details relating to a strategy for model building, the goodness of fit of the final model, or final model validation. The results from the Cox regression analyses were presented as HRs and associated 95% CIs in all publications.

The Cox PH model does not make assumptions about the shape of the underlying hazard function but does assume that the hazard rates for patient subgroups are proportional over time. Testing of the PH assumption was reported in two of the 28 publications detailing the use of Cox regression methods [7, 19]. Both publications reported that graphical methods were used to investigate proportionality by plotting cumulative hazard versus time, log (cumulative hazard) versus log (time) [7] or plots of Schoenfeld residuals versus log (-survival function) plots [19]. While neither these plots, nor the criteria for concluding that the PH was reasonable were presented, in both instances, the publications reported that the PH assumption was considered reasonable. An additional study stated that "Because the Cox proportional hazards model is the most commonly used approach to analyse time to event endpoints and because the two curves do not cross in this negative study, no tests for proportionality were done" [10]. However, an assessment of the survival curves reported in this publication revealed that the survival curves do cross at multiple points. A review of the survival curves found that in 20 of the 28 publications reporting the use of the Cox PH model, the survival curves of at least one of the survival outcomes crossed [3, 8, 10, 11, 13, 14, 16–18, 20, 22, 23, 25–30, 33, 34].

### Graphical display

All publications reported survival curves for all time to event endpoints analysed. The method of survival curve calculation across all publications was the KM method, although this was not always explicitly stated. Censored observations were marked on the survival curves in 21 of the publications, although these markings were rarely explained in the figure legends or publication text [3–7, 9–11, 14–16, 18, 20–22, 26, 27, 30, 31, 33, 34]. The patient numbers at risk were reported in 21 of the publications [5–7, 9–12, 14–28, 31–34]. All publications clearly distinguished between treatments in the survival curves, and the curves were described in the legends of half of the publications. Poor resolution and the use of relatively thick lines were the main limitations of the graphical survival displays—meaning that it was hard to distinguish points where treatments had very similar survival probabilities.

## Discussion

The use of valid and robust approaches in evidence-based medicine are crucial to clinical decision making. Survival analysis is a critical component of evidence-based medicine, particularly in the oncology setting, and has a huge potential to drive and impact decision making. The use of appropriate statistical methods are of key importance in survival analysis and are considered in this review.

### Reporting of analyses in general

The publications in this review represent the most recently published RCTs in some of the highest impact factor oncology journals. The majority of analyses present KM plots and the results from log-rank tests, and Cox regression analyses. The results of the review demonstrate that generally the studies were sufficient in terms of presenting the survival graphically but highlights serious deficiencies in terms of reporting the methods and validity of analyses.

Almost all publications analysed the ITT populations and there were no issues identified around unequal follow-up which could potentially bias analyses. While the majority of publications reported median follow-up, the method of calculation was generally unclear. Where reported, calculations of median follow-up were either based on all patients’ alive (survivors) or patients who have had an event. These methods can underestimate median follow-up time; a robust measure is considered to be based on the reverse KM estimator where the event indicator is reversed [35]. The lack of reporting means it is not possible to unequivocally determine whether median follow-up in the identified publications was calculated robustly.

The lack of endpoint definition in the identified publications is concerning—particularly in reference to secondary survival endpoints. A clear definition of each endpoint is essential to understanding the results of a study. Time to death can be considered unambiguous but endpoints such as time to progression may be less straightforward as disease progression is measured relative to baseline disease status and usually requires radiological assessment.

The review identified the applications of univariate and/or multivariate statistical models but the rationale for choice of analysis was rarely reported. Where both univariate and multivariate analyses were performed it was sometimes difficult to interpret which analysis results were being reported in the publications. In addition, publications rarely clarified the rationale for the choice of prognostic factors included in multivariate analyses. None of the Cox regression models reported aspects of the strategy for model building, the final model fit or any validations of the final model. Therefore readers have no option but to take the results of these analyses at face value and rely heavily on the assumption that the survival model used is the most appropriate choice and is a reasonable fit to the data.

### Cox regression

This review identified the failure of the majority of publications to report the validation of the Cox PH models in terms of the PH assumption. Estimates of treatment effect based on survival data that do not account for violation of the PH assumptions can be biased and depend on the length of follow-up in the study (the HR is not constant over time). A single study stated that the rationale for not performing tests for proportionality was based on the Cox PH model being the most commonly used and because the two curves did not cross [10]. This rationale is particularly alarming because the popularity of a particular method does not mean key assumptions of the model do not need to be validated, and survival curves that do not cross may still violate the PH assumption. In the current review, survival curves across publications detailing the use of the Cox regression model were assessed and in 71% of publications at least one survival curve included treatment arms that crossed—hence the PH assumption is likely to be violated. Without access to the IPD, the crossing of survival curves represents a crude method for assessing the PH assumption. Note that the survival curves do not necessarily need to cross for the PH assumption to be violated. The findings of this review could suggest that many publications use results based on models in which the key assumption was violated and consequently such results may be biased and inappropriate.

### Alternative statistical models

It is of particular interest that the current review failed to identify the use of parametric survival models or an alternative type of non-PH accelerated failure time (AFT) model [36]. Although rarely reported in publications the AFT model represents an alternative approach to PH models when the effects of treatment accelerate or delay the event of interest with no permanent effect in the context of the follow-up period [36]. An AFT model also allows the estimation of a time ratio which may be easier to interpret than a HR [36]. The results of the current review suggest that it is likely that Cox regression models are routinely chosen by trialists due to its widespread application, and to aid comparability with results of other trials [37]. The final choice of a statistical model for survival should be informed by goodness of model fit to a given dataset, and inappropriate statistical models may give results from which misleading conclusions are made.

### Previous work

To our knowledge, this work is the first to consider the reporting of survival analyses in clinical trials in terms of the potential implications for meta-analysis and HTA. The current review focused on survival curves and in particular the validity of Cox PH models. Previous work has reviewed survival analyses in cancer studies [38–40]. The first known review of publications of observational studies and RCTs reporting survival data highlighted presentational inadequacies of survival analyses published in cancer journals, and presented suggested guidelines to address these [38]. The latest work identified was essentially an update of the original Altman et al. review with the additional dimensions of also examining publications from other medical specialities in addition to oncology, and evaluating the reporting of survival analyses over time by comparing those published in 1991 and 2007 [39]. This study reported that, although the use of survival analyses continues to increase in the literature, noticeable reporting failures remain. In agreement with Altman et al., this study confirmed that a high proportion of articles are deficient in their reporting of survival analysis methods and results, and concludes there has been little improvement over the last decade [39]. An additional review of survival endpoints was restricted to RCTs and identified 125 Phase II or Phase III RCTs published in general and cancer related journals in 2004 [40]. This study represents the most comparable of the previous work to the current research presented, although in the previous work studies were restricted to those reporting survival endpoints as primary or secondary objectives of the study [40]. The study reported that all endpoints were totally defined in 52% of publications compared with 65% in our current research. The study also reported that the Cox model was used in 51% of articles compared with 88% in the current research. The study did not report other statistical models and therefore the current review reflects that more publications are reporting statistical models as opposed to log rank tests and simpler methods. The study did not identify the use of alternative parametric or AFT survival models [40].

### Implications of findings of review in terms of meta-analysis of survival outcomes

The failure of trialists to report survival endpoint definitions in sufficient detail has implications for the potential inclusion of study data into a meta-analysis. If investigators cannot determine endpoint definitions and the comparability of these across RCTs identified for inclusion into a meta-analysis then either RCTs with undefined endpoints may be omitted or additional assumptions regarding comparability may be required.

The PH assumption which underpins the most common strategy to the evidence synthesis of survival outcomes may in many cases be implausible thus impacting decisions based upon cost-effectiveness analyses. A potential alternative approach to single parameter meta-analysis of survival data is to perform the analysis based on time ratios obtained by modelling trial level data using the AFT model. Alternative approaches to meta-analysis of survival data based on multi-dimensional treatment effects as opposed to a single parameter (the HR) have been published in the literature [41–43]. Parametric survival functions are modelled and the difference in the parameters of these functions in a trial is considered the multidimensional treatment effect, which is synthesised and indirectly compared across trials. The parameters in the survival model regression are re-formulated to focus on the differences in the multiple parameters to understand the relative treatment effects. This method requires the digitisation of survival curves from primary publications which will require conservative assumptions around censoring or access to the IPD which in reality is unlikely to be feasible [44]. However, a method for the evaluation of consistency within NMA networks for this methodology has not yet been developed [45]. In terms of HTA, no information relating to the use of time ratios or multidimensional treatment effects for the meta-analysis of time to event data was identified in the guidelines for NICE, PBAC, IQWIG, CADTH or the National Centre for Pharmacoeconomics (NCPE).

All publications identified presented survival curves and whilst all of these clearly distinguished between treatment arms, 65% of the publications presented numbers at risk and censored observations. There are established methods to digitise the survival curve data and generate IPD from each of the publications which require conservative assumptions around censoring [44]. When performing conventional meta-analysis of survival data rather than take published study-level HRs from Cox regression analyses at face value investigators could explore the validity of PH assumptions using pseudo IPD. In addition, pseudo IPD can be used for the purposes of pursuing conventional single parameter meta-analysis (based on HRs or time ratios) or to investigate the feasibility of a multi-parameter NMA.

## Concluding Remarks

As with all systematic reviews, the current analysis was subject to limitations. A systematic review is only as robust as the data supporting it; therefore, a main limitation of this research was poor reporting regarding the statistical methods in the identified publications. Only English language publications were considered and there was no hand searching of grey literature. Only RCTs in clinical oncology were considered and the applicability of the results across other clinical areas is unknown. However, we have no reason to believe the findings of this review are not likely to be generalisable to the analysis of time to event endpoints in other clinical areas as it is a methodological issue not driven by the clinical context. The prevalence statistics obtained may be limited due to the relatively small sample of included studies (n = 32). An extension of the current study to a wider range of journals and clinical areas is required to achieve more reliable results. Empirical work to evaluate the validity of the time to event analyses of endpoints within the RCTs is beyond the scope of this review but further work to explore the suitability of the final models is recommended. The current work also highlights the importance of assessing the impact of the PH assumption violation on meta-analysis which could be achieved by conducting simulation studies.

The study presents a review of statistical approaches of survival analyses and the presentation of their results in clinical oncology Phase III RCTs. The date restrictions of the literature searches ensure that this review is representative of the most current practices of survival analyses in oncology. Trialists and statisticians are encouraged to explore the suitability of final survival models in terms of model fit and validation of the relevant assumptions, in particular the PH assumption and improve the quality of the reporting of their research. In terms of evidence synthesis, researchers are encouraged to carefully consider the validity of the methods from which single parameter estimates are derived. Where trial publications fail to comment upon the validity of the PH assumption, it is recommended that authors are contacted for clarification or a pseudo-level IPD is created to make an assessment. Where the PH is not reasonable, an alternative approach to evidence synthesis based on multi-dimensional treatment effects is recommended.

## Supporting Information

S1 Table Electronic literature searches. (DOCX)

S2 Table PRISMA checklist. (DOCX)
