# Supplementary material for: Review of the Reporting of Survival Analyses within Randomised Controlled Trials and the Implications for Meta-Analysis
Source: PLoS One. 2016 May 5;11(5):e0154870. doi: 10.1371/journal.pone.0154870 (PMC4858202; doi:10.1371/journal.pone.0154870)
Supplement: S1 Table — (DOCX) [file pone.0154870.s001.docx]

**Embase** 1980 to 2015 Week 34

Accessed 28/08/2015

[TL_Altman_Update]

| **#** | **Searches** | **Results** |
| --- | --- | --- |
| 1 | "journal of clinical oncology".jn. | 55043 |
| 2 | ca a cancer journal for clinicians.jn. | 1742 |
| 3 | lancet oncology.jn. | 1802 |
| 4 | cancer discovery.jn. | 1714 |
| 5 | "journal of the national cancer institute".jn. | 18295 |
| 6 | 1 or 2 or 3 or 4 or 5 | 78596 |
| 7 | (random$ or placebo$ or single blind$ or double blind$ or triple blind$).ti,ab. | 1101223 |
| 8 | RETRACTED ARTICLE/ | 7620 |
| 9 | 7 or 8 | 1108661 |
| 10 | (animal$ not human$).sh,hw. | 3819238 |
| 11 | (book or conference paper or editorial or letter or review).pt. not exp randomized controlled trial/ | 4198740 |
| 12 | (random sampl$ or random digit$ or random effect$ or random survey or random regression).ti,ab. not exp randomized controlled trial/ | 64928 |
| 13 | 9 not (10 or 11 or 12) | 849388 |
| 14 | 6 and 13 | 10379 |
| 15 | limit 14 to dd=20150401-20150731 | 238 |

**Ovid MEDLINE(R) In-Process & Other Non-Indexed Citations and Ovid MEDLINE(R)** 1946 to Present

Accessed 28/08/2015 [TL_Altman_Update_Medline]

| **#** | **Searches** | **Results** |
| --- | --- | --- |
| 1 | "journal of clinical oncology".jn. | 21700 |
| 2 | ca a cancer journal for clinicians.jn. | 2079 |
| 3 | lancet oncology.jn. | 4713 |
| 4 | cancer discovery.jn. | 1267 |
| 5 | "journal of the national cancer institute".jn. | 20714 |
| 6 | 1 or 2 or 3 or 4 or 5 | 50473 |
| 7 | "randomized controlled trial".pt. | 408576 |
| 8 | (random$ or placebo$ or single blind$ or double blind$ or triple blind$).ti,ab. | 881394 |
| 9 | (retraction of publication or retracted publication).pt. | 8088 |
| 10 | 7 or 8 or 9 | 975493 |
| 11 | (animals not humans).sh. | 4001326 |
| 12 | ((comment or editorial or meta-analysis or practice-guideline or review or letter or journal correspondence) not "randomized controlled trial").pt. | 3544190 |
| 13 | (random sampl$ or random digit$ or random effect$ or random survey or random regression).ti,ab. not "randomized controlled trial".pt. | 55178 |
| 14 | 10 not (11 or 12 or 13) | 727634 |
| 15 | 6 and 14 | 5277 |
| 16 | limit 15 to ed=20150401-20150731 | 99 |
